# Antifungal activity of non-conventional yeasts against *Botrytis cinerea* and non*-Botrytis* grape bunch rot fungi

**DOI:** 10.3389/fmicb.2022.986229

**Published:** 2022-08-23

**Authors:** Evelyn Maluleke, Neil Paul Jolly, Hugh George Patterton, Mathabatha Evodia Setati

**Affiliations:** ^1^Department of Viticulture and Oenology, South African Grape and Wine Research Institute, Stellenbosch University, Matieland, South Africa; ^2^Post Harvest and Agro-Processing Technologies, ARC Infruitec-Nietvoorbij (The Fruit, Vine and Wine Institute of the Agricultural Research Council), Stellenbosch, South Africa; ^3^Centre for Bioinformatics and Computational Biology, Stellenbosch University, Matieland, South Africa

**Keywords:** *Wickerhamomyces anomalus*, antagonistic yeasts, biological control, cell wall lytic enzymes, volatile organic compounds

## Abstract

Grapes harbour a plethora of non-conventional yeast species. Over the past two decades, several of the species have been extensively characterised and their contribution to wine quality is better understood. Beyond fermentation, some of the species have been investigated for their potential as alternative biological tools to reduce grape and wine spoilage. However, such studies remain limited to a few genera. This work aimed to evaluate the antagonistic activity of grape must-derived non-conventional yeasts against *Botrytis cinerea* and *non-Botrytis* bunch-rotting moulds and to further elucidate mechanisms conferring antifungal activity. A total of 31 yeast strains representing 21 species were screened on different agar media using a dual culture technique and liquid mixed cultures, respectively. *Pichia kudriavzevii* was the most potent with a minimum inhibitory concentration of 10^2^ cells/mL against *B. cinerea* but it had a narrow activity spectrum. Twelve of the yeast strains displayed broad antagonistic activity, inhibiting three strains of *B. cinerea* (B05. 10, IWBT FF1 and IWBT FF2), a strain of *Aspergillus niger* and *Alternaria alternata.* Production of chitinases and glucanases in the presence of *B. cinerea* was a common feature in most of the antagonists. Volatile and non-volatile compounds produced by antagonistic yeast strains in the presence of *B. cinerea* were analysed and identified using gas and liquid chromatography mass spectrometry, respectively. The volatile compounds identified belonged mainly to higher alcohols, esters, organosulfur compounds and monoterpenes while the non-volatile compounds were cyclic peptides and diketopiperazine. To our knowledge, this is the first report to demonstrate inhibitory effect of the non-volatile compounds produced by various yeast species.

## Introduction

Grape berries harbour a complex microbial community comprising myriads of yeast, bacterial and mould species, that play pivotal roles in grape quality and wine production (Varela and Borneman, 2017; Liu et al., 2020; Griggs et al., 2021). Within this community, some environmental bacteria as well as moulds are known to flourish on grape berries (Barata et al., 2012). Moulds may exist on grapes either as saprophytes and opportunistic pathogens or as obligate parasites. For instance, *Botrytis cinerea*, *Rhizopus* spp., *Penicillium* spp., *Aspergillus* spp., as well as *Alternaria alternata* are associated with grape rot. *B. cinerea* is a widely described causative agent of grey rot while *Aspergillus niger* and *A. alternata* are associated with black rot (Steel et al., 2013). Yeasts, lactic acid bacteria as well as acetic acid bacteria influence wine aroma and flavour. In particular, yeasts are key drivers of the alcoholic fermentation process, however, some basidiomycetous yeasts such as species of the genera *Rhodotorula*, *Cryptococcus*, *Filobasidium*, *Sporobolomyces* as well as ascomycetous yeasts of the genera *Candida*, *Metschnikowia*, *Zygoascus* and *Pichia* which are oxidative or weakly fermentative do not thrive in a wine fermentation environment (Barata et al., 2012).

Grape and grape must-associated yeasts have widely been studied to decipher their role in wine fermentation and their contribution to wine organoleptic properties. Such studies have resulted in commercialisation of non-*Saccharomyces* yeasts including *Lachancea thermotolerans*, *Torulaspora delbrueckii*, *Metschnikowia pulcherrima* and *Pichia kluyveri* to name a few (Vejarano and Gil-Calderón, 2021). However, more than 40 yeast species have been isolated from grape and wine fermentation environments (Jolly et al., 2014) and recent studies show that some of these have great potential as bioprotectants and biocontrol agents against spoilage organisms (Kuchen et al., 2019; Mewa-Ngongang et al., 2019a) and phytopathogens (Bleve et al., 2006; Nally et al., 2015; Cordero-Bueso et al., 2017; Mewa-Ngongang et al., 2019b; Marsico et al., 2021; Sabaghian et al., 2021). The most frequently reported yeast antagonists include mainly strains belonging to the *Metschnikowia pulcherrima*, *Hanseniaspora* spp., *Pichia* spp. e.g., teleomorph *Meyerozyma* (*Pichia*) *guilliermondii*, as well as strains of *Wickerhamomyces anomalu*s, *Aureobasidium pullulans* (yeast-like fungus) and *Meyerozyma guilliermondii* (Parafati et al., 2015; di Francesco et al., 2016; Cordero-Bueso et al., 2017; Chen et al., 2018; Pereyra et al., 2021).

Yeasts have been shown to express various mechanisms in antagonistic interactions. These include competition for space and nutrient (Parafati et al., 2015), secretion of extracellular lytic enzymes such as protease, glucanase and chitinase (Cordero-Bueso et al., 2017; Agarbati et al., 2022) and volatile organic compounds (Fredlund et al., 2004; Cordero-Bueso et al., 2017; Mewa-Ngongang et al., 2019b). Amongst the VOCs esters and higher alcohols such as ethyl acetate, phenyl acetate, isoamyl alcohol, benzyl alcohol, isoamyl octanoate, 2-methyl-1-propanol and 2-phenylethanol, have been implicated in inhibitory activities. These compounds are reported to suppress conidia germination and mycelium growth of *B. cinerea* and most of *Penicillium* spp. under both *in vitro* and *in vivo* conditions (Chen et al., 2018; Oro et al., 2018; Choińska et al., 2020; Piasecka-jo and Choin, 2020; Yalage Don et al., 2020). Antifungal activity is however strain dependent and therefore necessitates a screening of a myriad of isolates and strains of different origins in order to find strains with broad specificity.

Several biocontrol agents or products such as Aspire^®^ (*Candida oleophila*), Candifruit^®^ (*Candida sake*), Shemer^®^ (*Metschnikowia fructicola*) and BoniProtect^®^ (*Aureobasidium pullulans*) consisting of yeast or yeast-like fungi as active ingredients have been registered and made it to the market (Pretscher et al., 2018; Freimoser et al., 2019; Zhang et al., 2020). However, sustainable production and application of some of these were not realised and they were ultimately withdrawn from the market (Zhang et al., 2020). Some of the key limitations for wide use of biocontrol agents include narrow spectrum of activity, reduced efficacy in commercial and field conditions (Zhang et al., 2020). Traditionally, grey rot and other grapevine pathogens are controlled with the use of synthetic fungicides which have preventive and curative effects. While these fungicides have sustained grape and wine production for centuries, their application in vineyards may result in environmentally harmful residues (Ons et al., 2020; Schusterova et al., 2021). Moreover, application of such fungicides is not permissible within a 30-day period prior to harvest (Abbey et al., 2019; Ons et al., 2020; Zhang et al., 2020). Consequently, integrating grape and must derived yeasts with antifungal activity against several phytopathogens, to complement routine vineyard spray programs remains an attractive alternative that deserves in-depth exploration (di Canito et al., 2021; Lahlali et al., 2022).

Over the past decade, a wide range of oxidative and weakly fermentative non-conventional yeasts have been isolated and identified from South African vineyards (Setati et al., 2012; Bagheri et al., 2015; Ghosh et al., 2015; Shekhawat et al., 2018). While some of the isolates have been evaluated for their oenological traits (Rossouw and Bauer, 2016; Rollero et al., 2018; Porter et al., 2019), their biotechnological potential remains largely untapped. This study aimed to unravel the antifungal traits of non-conventional yeasts derived from wine grapes and must. The expression of antifungal activity *in vitro* and on grape berries was assessed.

## Materials and methods

### Microbial strains and culture media

Yeast strains isolated from grape must were obtained from the culture collection of the South African Grape and Wine Research Institute (SAGWRI), Stellenbosch University. Thirty-one strains (Supplementary Table S1) were routinely grown and maintained on Wallerstein Nutrient (WLN) agar (Merck Millipore, South Africa). For long-term storage, the strains were stored at −80°C in 25% (*v*/*v*) glycerol in cryogenic tubes. Three strains of *B. cinerea*, laboratory strain (B05. 10), grape strains (IWBT FF1 and IWBT FF2) isolated from Cabernet Sauvignon grapes obtained from Thelema Mountain vineyard, South Africa (33°54′46.1′S 18°56′30.7′E), one strain of *Aspergillus niger* and *Alternaria alternata* isolated from soil collected from Stellenbosch University’s Welgevallen experimental farm (33°57′03.0′S 18°52′05.6′E), were used in this study. Filamentous fungal cultures were revived on Malt Extract agar (MEA; Merck Millipore, South Africa) containing 2% (*w*/*v*) bacteriological agar. Yeast inoculums were prepared from overnight cultures grown in 5 ml YPD broth containing per litre (10 g yeast extract, 20 g peptone and 20 g glucose). Fresh yeast cultures were collected by centrifugation at 10,625 *g* for 5 min and washed twice with sterile 0.9% (*w*/*v*) NaCl solution. The yeast suspensions were adjusted to OD_600_ 0.1 (≈ 10^6^ CFU/ml) using 0.9% (*w*/*v*) NaCl.

### *In vitro* screening for yeast antagonistic activity

#### Dual culture plate assay

**Figure 1:**
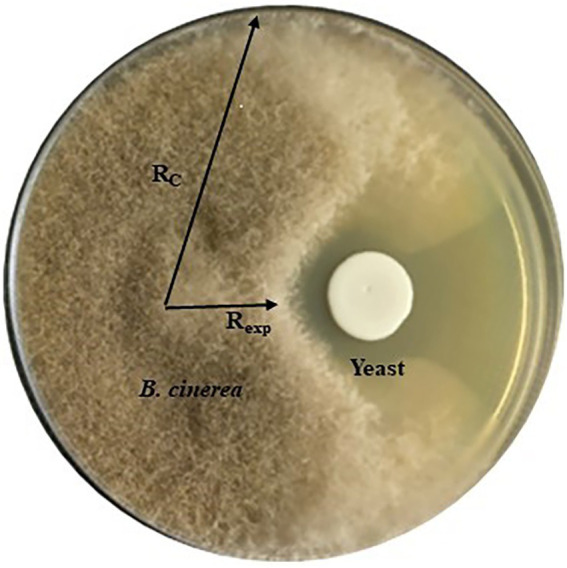
A representation of the dual culture assay showing yeast antifungal activity against *Botrytis cinerea* and how the inhibition was measured. *R*_c_ represents the longest distance of fungal mycelium growth from the inoculated fungal plug and *R*_exp_ is the horizontal distance from the inoculated fungal plug towards the yeast colony.

Antifungal activity of 31 yeast strains (Supplementary Table S1) was tested against three strains of *B. cinerea* (B05. 10, IWBT FF1) and IWBT FF2, *A. niger* (IWBT FF3) and *A. alternata* (IWBT FF4) by *in vitro* assay on MEA and low glucose (0.2% *w*/*v*) YPD agar referred to as YPD-L from hereon. Three replicate plates were prepared for each yeast. For the screening, 6 × 6 mm^2^ agar plugs of the filamentous fungi were obtained from the margins (active growth zone) of young mycelia grown on MEA and inoculated face-down onto fresh agar at a distance of 27 mm away from the Petri dish edge (Standard 90 mm Petri dishes, Sigma-Aldrich, South Africa). Twenty microliter of yeast suspension (≈ 10^6^ cells/ml) was inoculated 30 mm away from the fungal plug on the opposite side of the plate. Negative control plates were only inoculated with the filamentous fungi. A strain of the yeast-like fungus *Aureobasidium pullulans* W32 was used as a positive control as multiple strains of this fungus have been shown to inhibit *B. cinerea* (Yalage Don et al., 2020). The plates were incubated for 5 days at 25°C in the dark for *B. cinerea* strains experiments and under standard light conditions for *A. niger* and *A. alternata*. Mycelial growth was observed and images were captured to record the growth. The inhibition percentage was calculated as [(*R*_c_–*R*_exp_)/*R*_c_] × 100%, where *R*_c_ represents the longest distance of fungal mycelium from the inoculated fungal plug and *R*_exp_ is the horizontal distance from the inoculated fungal plug towards the yeast colony, which shows the inhibitory effect (Chen et al., 2018; Figure 1).

#### Liquid co-culture assay

To determine whether yeast antifungal activity varied between culture conditions, liquid co-culture assays were conducted. Ten microliter of yeast suspension (≈ 10^6^ cells/mls) and of the spore suspension (≈ 10^6^ spores/ml) of each pathogen (*B. cinerea*, *A. niger* and *A. alternata*) were inoculated into 24-well plates containing 2 ml of malt extract broth. The cultures were incubated at 25°C for 5 days. Microscopic observations of 20 μl wet mounts, were conducted to assess hyphal formation. Five fields on each slide were assessed and images were captured at 40, 100 and 400*x* magnification.

After the initial screening, yeast species with antifungal activity against the tested pathogens were selected and investigated for their Minimum inhibiting concentration (MIC) and production of cell wall lytic enzymes.

### Determining modes of action

#### Evaluation of minimum inhibiting concentration

Minimum yeast cell concentration necessary to inhibit the growth of *B. cinerea* was investigated on yeast strains that displayed inhibition on plates and in liquid co-culture conditions against all the pathogens tested in this study. Fresh yeast cultures were inoculated in YPD broth and grown overnight at 25°C. For each yeast strain tested, malt extract broth and YPD agar plates were inoculated with cell suspensions from 10^2^ to 10^6^ cells/ml. Ten microliter of *B. cinerea* IWBT FF1 spore suspensions (~ 10^6^ spores/ml) were inoculated on the centre of the Petri dish and into 2 ml malt extract broth in 24-well plates. A negative control with *B. cinerea* spore suspension was also prepared. The plates were incubated at 25°C for 5 days. The results were considered positive when no or limited *B. cinerea* mycelial formation was observed. The experiment was conducted in three replicates. The MIC was recorded as the lowest yeast cell concentration required to inhibit *B. cinerea* mycelial growth.

#### Screening yeasts for chitinase and glucanase production

##### Preparation of colloidal chitin

Colloidal chitin was prepared according to the method described by Agrawal and Kotasthane (2012). Briefly, 20 g of chitin (chitin from shrimp cells-Sigma-Aldrich, South Africa) was dissolved in 350 ml cold 12 M HCl overnight at 4°C with continuous mixing on a magnetic stirrer plate, followed by extraction with 2,000 ml of ice-cold 95% ethanol and an overnight incubation at 25°C. The precipitate was centrifuged at 1,479 *g* for 20 min at 4°C. The pellet was washed with sterile distilled water three times and centrifuged at 1,479 *g* for 5 min at 4°C till the acid and ethanol were completely washed-off. The colloidal chitin obtained had a soft, pasty consistency with 90–95% moisture and was stored at 4°C until further use.

##### Hydrolytic enzyme production

In order to determine if the yeasts in the current study produced chitinases and β-1,3-glucanases as part of their antagonistic activity, dual culture plate assay were performed on agar supplemented with appropriate substrates. The protocol was adopted from Chen et al. (2018) and was adjusted by excluding the mineral salts from the media composition. Chitinase production was determined on YPD-L agar supplemented with 0.45% (*w*/*v*) colloidal chitin and 0.15 g/l bromocresol purple. The medium was adjusted to pH 4.7 with 1 M HCl and autoclaved. Glucanase activity was screened on a medium containing 0.5% (*w*/*v*) yeast extract, 1% (*w*/*v*) peptone and 0.2% (*w*/*v*) laminarin (Merck Millipore, South Africa), medium was adjusted to pH 4 with HCl (1 M) and mixed with bacteriological agar to a final concentration of 2% (*w*/*v*) after autoclaving (Ghosh et al., 2015). The plates were inoculated and incubated as described in the *in vitro* dual assay. Chitinase activity was detected by the formation of a purple zone around the yeast colony, while glucanase activity was visualised by staining the laminarin plates with 0.1% (*w*/*v*) Congo red for 1 h, followed by de-staining with 1 M NaCl till a clear zone around the yeast colony was observed (Ghosh et al., 2015; Chen et al., 2018). The inhibition percentage was measured using [(*R*_c_–*R*_exp_)/*R*_c_] × 100%. All the measurements were done on triplicate plates. Enzyme production was recorded as either weak or strong depending on the inhibition percentage (Table 1).

**Table 1:** Exemplar images of the chitinase and glucanase plate screening showing yeast and *Botrytis cinerea* growth, enzyme production and weak or strong inhibition zone.

Enzyme	
Chitinases	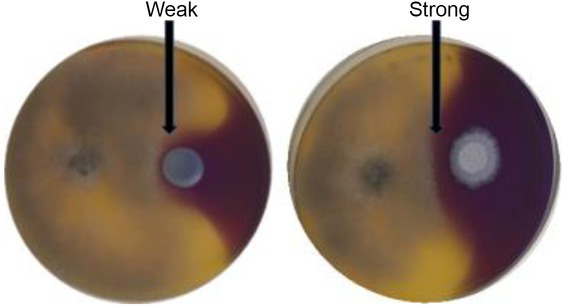
β-1,3-Glucanases	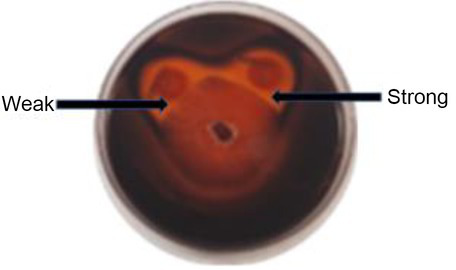

#### Hyphal structure phenotype of *Botrytis cinerea*

*Botrytis cinerea* and yeasts co-cultured for 5 days at 25°C on YPD-L agar were directly used for confocal microscopy analysis. After incubation, all the mycelium directly next to the inhibition zone were collected into an Eppendorf tube and washed with 1 ml phosphate-buffered saline (PBS; pH 7.4; Na_2_HPO_4_; Merck Millipore, South Africa). To measure chitin level, the hyphae were stained with 10 μl calcofluor white (Merck Millipore, South Africa) after the addition of 10 μl of 10% KOH (Merck Millipore, South Africa). For glucan content, the hyphae were stained with trypan blue (Merck Millipore, South Africa). Z-sectioning image acquisition was performed on a Carl Zeiss confocal laser scanning microscope (LSM) 780 Elyra S1 with super resolution structured illumination microscopy (SR-SIM super resolution) platform. Z-series images were taken at 0.5 μm intervals through the specimens. The excitation laser used was the violet laser with 407-nm wavelength, and the emission filter used was the Pacific Blue channel with a 450/40 band-pass filter for calcofluor white and trypan blue stained cells. Images were processed and background subtracted using the Zeiss Zen lite 2011 software and presented in a maximum-intensity projection.

For further investigation, yeasts with broad spectrum activity were selected and analysed for the production of VOCs and non-volatile compounds.

#### Volatile organic compounds production

**Figure 2:**
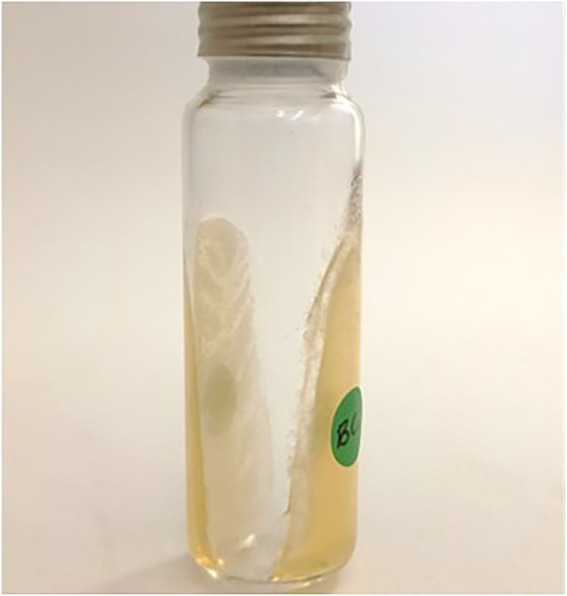
Headspace vial with YPD-L agar for automated sampling of volatile organic compounds produced by antagonistic yeasts in the presence of *Botrytis cinerea* IWBT FF1.

Experimental set up for SPME automated sampling of VOCs and analysis was done according to Yalage Don et al., 2020. Two layers of sterile YPD-L (1.5 ml) were prepared by pouring 1.5 ml of the agar on opposite sides of the vial as illustrated in Figure 2. For inoculation, *B. cinerea* IWBT FF1 spore suspension was prepared in sterile distilled water (10^6^ spores/ml, 10 μl) and spread on one side of the vial using a 10 μl inoculation loop (LP ITAKIAN SPA, Milan, Italy) then incubated for 48 h at 25°C, after which the yeast cell suspension (≈ 10^6^ cells/ml, 10 μl) prepared in 0.9% (*w*/*v*) NaCl was spread on the opposite side of the vial. The vials were incubated for a further 10 days. Control vials were inoculated with either *B. cinerea* spore suspension or a yeast cell suspension. Four replicates were performed for each antagonist-pathogen combination and controls.

##### Sample preparation for VOCs analysis (HS-SPME–GC–MS)

Prior to GC–MS analysis, 50 μl of a 10 ppm Anisole d8 solution was added to the centre of each vial as an internal standard. The vials were incubated in the autosampler at 50°C for 5 min, after which a 50/30 μm divinylbenzene/carboxen/polydimethylsiloxane (DVB/CAR/PDMS) SPME fibre (Supelco, Bellafonte, PA, United States) was exposed to the headspace of the vial for 30 min at the same temperature. After equilibration, the fibre was injected onto the injector at 250°C, and 10 min were allowed for desorption of the compounds.

##### Chromatographic conditions

Analysis of VOCs was performed on an Agilent Gas Chromatography, model 6,890 N (Agilent, Palo Alto, CA, United States), coupled with an Agilent mass spectrometer detector (MS), model 5975B Inert XL EI/CI (Agilent, Palo Alto, CA, United States) equipped with a CTC Analytics PAL autosampler. The chromatographic separation of compounds was performed on a polar J&W DB-FFAP (60 m, 0.25 mm i.d., 0.5 μm film thickness) capillary column. The oven temperature program was set as follows: 40°C held for 1 min, then ramped up to 150°C at 25°C/min and held for 3 min, and again ramped up to 200°C at 5°C/min and held for 5 min, and finally up to 250°C at 5°C/min and held there for 2 min. The total run time was 30.54 min. Helium at a constant flow rate of 1.0 ml/min was used as a carrier gas. The injector operated in a split-less mode maintained at 250°C throughout the analysis. Both the purge flow and gas saver flow were activated at 50 ml/min for two and 5 min, respectively. The MS-detector was operated in single ion monitoring (SIM) mode. The ion source and quadrupole temperatures were maintained at 230°C and 150°C, respectively, with the transfer line set at 250°C. Compounds were identified using GC–MS retention times and cross-referencing their mass spectra with the NIST05 spectral library.

#### Extraction and analysis of non-volatile compounds

Non-volatile metabolites were extracted following a method described by Sasidharan et al. (2012). The antagonist yeast was co-cultured with *B. cinerea* IWBT FF1 in a 24-well plate containing 2 ml malt extract broth. Controls were prepared by growing *B. cinerea* and the yeasts in monocultures. Cultures were incubated at 25°C for 5 days. After incubation, 0.5 ml samples were collected and transferred into 0.5 ml of freshly prepared N-ethylmaleimide-methanol (NEM) solution (4 mM) kept in 1.5 ml screw cap tubes equilibrated using dry ice. The cultures were pelleted by flash centrifugation for 2 min (20,000 *g*; −9°C) and the supernatant containing the extracellular metabolites was transferred into new microcentrifuge tubes and stored at −80°C till analysis. Prior to analysis, samples were transferred to an LC vial, and all the vials were stored at −80°C for LC–MS analysis.

##### Liquid chromatography–mass spectrometry analysis

A Waters Synapt G2 Quadrupole time-of-flight (QTOF) mass spectrometer (MS) connected to a Waters Acquity ultra-performance liquid chromatograph (UPLC; Waters, Milford, MA, United States) was used for high-resolution UPLC-MS analysis. Column eluate first passed through a Photodiode Array (PDA) detector before going to the mass spectrometer, allowing simultaneous collection of UV and MS spectra. Electrospray ionisation was used in negative mode with a cone voltage of 15 V, desolvation temperature of 275°C, desolvation gas at 650 l/h, and the rest of the MS settings optimised for best resolution and sensitivity. Data were acquired by scanning from m/z 150 to 1,500 m/z in resolution mode as well as in MSE mode. In MSE mode two channels of MS data were acquired, one at a low collision energy (4 V) and the second using a collision energy ramp (40–100 V) to obtain fragmentation data as well. Leucine enkephalin was used as lock mass (reference mass) for accurate mass determination and the instrument was calibrated with sodium formate. Separation was achieved on a Waters HSS T3, 2.1 × 100 mm, 1.7 μm column. An injection volume of 2 μl was used and the mobile phase consisted of 0.1% formic acid (solvent A) and acetonitrile containing 0.1% formic acid as solvent B. The gradient started at 100% solvent A for 1 min and changed to 28% B over 22 min in a linear way. It then went to 40% B over 50 s and a wash step of 1.5 min at 100% B, followed by re-equilibration to initial conditions for 4 min. The flow rate was 0.3 ml/min and the column temperature was maintained at 55°C. Compounds were quantified in a relative manner against a calibration curve established by injecting a range of catechin standards from 0.5 to 100 mg/l catechin. Data was processed using MSDIAL and MSFINDER (RIKEN Centre for Sustainable Resource Science: Metabolome Informatics Research Team, Kanagawa, Japan).

### Grape bioassay

For the *in vivo* test, yeast strains that proved to be the most effective antagonists against all the pathogens investigated in this study were selected and assessed for their Inhibitory activity on grapes. Early sweet seedless white grapes (*Vitis vinifera*) were obtained from a local supermarket (Stellenbosch). Grape berries surface was disinfected by soaking grapes in 1% (*v*/*v*) sodium hypochlorite solution for 5 min and rinsed three times with sterile distilled water. The water was allowed to dry prior to the next step. Grapes were uniformly wounded with a sterile needle (< 1 mm diameter per wound) and allowed to dry prior to yeast treatments. Yeast cell and spore suspensions were prepared as previously described. For preventive treatments, wounded grapes were inoculated with 20 μl (≈ 10^6^ cells/ml) of various yeast suspension using a micropipette and incubated 24 h at 25°C. Subsequently, the berries were inoculated with 20 μl (≈ 10^6^ spores/ml) of *B. cinerea* IWBT FF1. The berries were incubated at 25°C and to maximise the attainment of a higher level of humidity, a heavily wet piece of paper towels was placed in each closed airtight container (Dixie injection & Blow moulders, South Africa). Negative controls (six berries each) were prepared by inoculating the fungal spores on the wounded berries without yeast cells. The antagonistic properties of the selected yeast species were analysed visually by assessing the grape colour changes and fungal development on treated berries. The disease severity was evaluated by a visual score of 1-to-4 (1: no visible symptoms; 2: soft rot; 3: formation of mycelium; 4: sporulation of mould).

### Statistical analysis

Data are expressed as mean ± standard deviation (SD). The significance of differences between each experiment and control was determined in XLSTAT software. *p* ≤ 0.05 was considered statistically significant (Data analysis and Statistical Solution for Microsoft Excel, Addinsoft, Paris, France 2022).

## Results

### Mycelial growth inhibition and cell wall hydrolases

**Figure 3:**
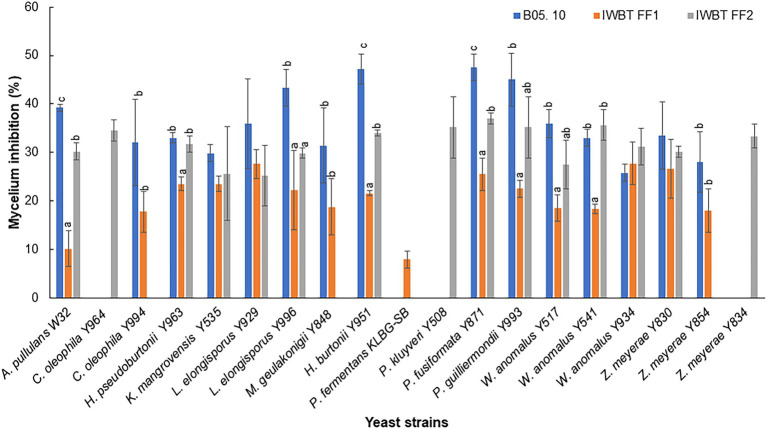
Yeast inhibition of *Botrytis cinerea* B05. 10, IWBT FF1 and IWBT FF2 mycelial growth. Bars represent average inhibitions of three replicates with standard deviation. Different superscript letters (^a, b, c^) show significant differences according to Tukey and Dunnett test (*p* <  0.05) when comparing the mycelium inhibition of each *B. cinerea* strain by different yeasts. Strains with no superscript letters indicate those with no significant difference.

Thirty-one yeast strains isolated from grape must were obtained from the SAGWRI culture collection and screened for antifungal activity against *B. cinerea*, *A. niger* and *A. alternata*. Twenty-three strains representing 15 species displayed varying inhibitory activity against the fungal pathogens in dual plate assays (Table 2). All the antagonistic strains also inhibited growth of the filamentous fungi in liquid cultures. This was evidenced by absence or limited formation of hyphae in the cultures when visualised under the microscope (Supplementary Figure S1). Among the 23 strains, 12 exhibited broad antagonistic activity inhibiting three strains of *B. cinerea*, as well as the strain of *A. niger* and *A. alternata*, while 11 had narrow-spectrum activity. Higher inhibition percentages were mostly recorded against *B. cinerea* B05.10 than the two grape strains (Figure 3). Furthermore, within the species, different yeast strains exhibited varying inhibition capabilities. For instance, among the *Wickerhamomyces anomalus* strains, Y934 displayed similar inhibition levels for all *B. cinerea* strains, while Y517 and Y541 were more inhibitory against B05.10 and IWBT-FF2. Similarly, intraspecific variabilities were observed with *Candida oleophila* and *Zygoascus meyerae* (Figure 3). *Pichia kudriavzevii* Y508 exhibited the lowest MIC of 10^2^ cells/ml followed by *W. anomalus* strains (Y541, Y517 and Y934) and *C. oleophila* Y964 at 10^3^ cells/ml, while for most strains an MIC of 10^4^ cells/ml was observed (Table 2).

**Table 2:** Antifungal activity phenotypes of yeast species and strains.

Yeasts strains	Inhibition spectrum	% Mycelial inhibition against B05.10	Inhibitory activity in liquid cultures	Minimum inhibiting concentration (MIC)	Chitinase	s
*A. pullulans*	+++	39.22	+	ND	Strong	Strong
*C. azyma* Y979	---	−	−	ND	ND	ND
*C. apicola* Y957	---	−	−	ND	ND	ND
*C. lusitaniae Y833*	---	−	−	ND	ND	ND
*C. oleophila* NOVA-CH	++−	−	+	10^4^	Weak	Strong
*C. oleophila* Y964	++−	−	+	10^3^	Strong	ND
*C. oleophila* Y994	+++	32.02	+	10^4^	Weak	Weak
*F. capsuleginum* Y938	---	−	−	ND	ND	ND
*H. pseudoburtonii* Y963^**^	+++	32.94	+	10^5^	Strong	Strong
*K. mangrovensis* Y535	++−	29.83	+	ND	ND	ND
*L. elongisporus* Y929^**^	+++	35.94	+	10^5^	Weak	Strong
*L. elongisporus* Y996^**^	+++	43.27	+	10^5^	Weak	Strong
*M. chrysoperlae* Y955	++−	−	+	ND	ND	ND
*M. bicuspidata* Y540	---	−	−	ND	ND	ND
*M. geulakonigii* Y848	+++	31.39	+	10^5^	Weak	Strong
*H. burtonii* Y951	+++	47.17	+	10^4^	Strong	Strong
*P. fermentans* Y995	++−	−	+	10^4^	ND	ND
*P. fermentans* KLBG-SB	++−	−	+	10^4^	ND	ND
*P. guilliermondii* Y993	+++	44.99	+	10^6^	Strong	Strong
*P. kluyveri* FRU-1	---	−	−	ND	ND	ND
*P. kluyveri* NOVA-CH	---	−	−	ND	ND	ND
*P. kluyveri* SIL-1	---	−	−	ND	ND	ND
*P. kudriavzevii* Y508	++−	−	+	10^2^	Weak	ND
*P. manshurica* Y510	++−	−	+	10^5^	Weak	ND
*P. occidentalis* BGLD-CH	++−	−ss	+	10^4^	Weak	ND
*P. fusiformata* Y871	+++	47.54	+	10^6^	Weak	Weak
*W. anomalus* Y517 ^**^	+++	35.98	+	10^3^	Strong	Weak
*W. anomalus* Y541^**^	+++	32.95	+	10^3^	Strong	Strong
*W. anomalus* Y934^**^	+++s	25.77	+	10^3^	Strong	Weak
*Z. meyerae* Y830	++−	33.98	+	10^5^	Weak	Strong
*Z. meysserae* Y834	++−	−	+	10^5^	Strong	Strong
*Z. meyerae* Y854	++−	28.07	+	10^5^	Weak	Strong

Key: (Inhibition spectrum) +++, ++− or --- denotes strains capable of inhibiting all the three pathogens, two pathogens and no activity, respectively. Chitinase and glucanase activity: Strong, Weak or ND denotes inhibition percentage > 50% and < 50% and not determined, respectively.

Yeast strains marked with ^**^ were selected for further investigation.

Following the initial screening on agar plates and liquid media, yeasts that inhibited the growth of all the pathogens were selected and investigated for the production of cell wall hydrolytic enzymes, volatile and non-volatile compounds. Overall, inter- and intra-specific variations in chitinase and glucanase production was observed (Table 2). For instance, within the genus *Pichia*, strong chitinase-associated inhibition was evident in *Hyphopichia burtonii* (formerly *Pichia burtonii*) and *P. guilliermondii*, while in *P. kudriavzevii*, *P. manshurica* and *P. occidentalis* this activity was weak. Within the *W. anomalus* species, all strains exhibited strong chitinase activity but only strain Y541 exhibited strong glucanase activity. Similarly, with *Zygoascus meyerae*, two strains displayed weak chitinase while all three strains had strong glucanase activity.

Selected yeast strains were co-cultured with *B. cinerea* and the hyphal chitin and glucan levels were assessed using a confocal microscope following staining with calcofluor white and trypan blue. Overall, huge variations were observed in the hyphal chitin and glucan levels, but tendentially a decrease was evident in the hyphae from the mycelia exposed to the antagonistic yeast (Table 3). In particular, *B*. *cinerea* hyphae from the mycelia exposed to *W. anomalus* Y541 and *W. anomalus* Y934 showed significant reduction in chitin levels, while a significant reduction in glucan levels was observed in the mycelia exposed to *W. anomalus* Y541 and *W. anomalus* Y517.

**Table 3:** Chitin and glucan content in *Botrytis cinerea* hyphae treated with various yeast strains capable of producing chitinase and glucanase.

Cultures	Chitin level	Glucan level
*B. cinerea*	25.22 ± 132	91.78 ± 30.21
*B. cinerea* + *W. anomalus* Y541	18.76 ± 6.03^**^	28.63 ± 9.11^**^
*B. cinerea* + *W. anomalus* Y517	18.26 ± 10.66	48.66 ± 27.29^**^
*B. cinerea* + *W. anomalus* Y934	43.02 ± 8.33^**^	44.12 ± 14.98
*B. cinerea* + *H. pseudoburtonii* Y963	19.18 ± 8.92	50.77 ± 36.31
*B. cinerea* + *L. elongisporus* Y929	12.63 ± 4.26	59.36 ± 49.46
*B. cinerea* + *L. elongisporus* Y996	28.17 ± 9.92	62.85 ± 41.13

Data expressed as mean ± SD.

** Show significant difference between yeast treatment and the control according to *t*-test (*p* < 0.05).

### Volatile organic compounds production

**Figure 4:**
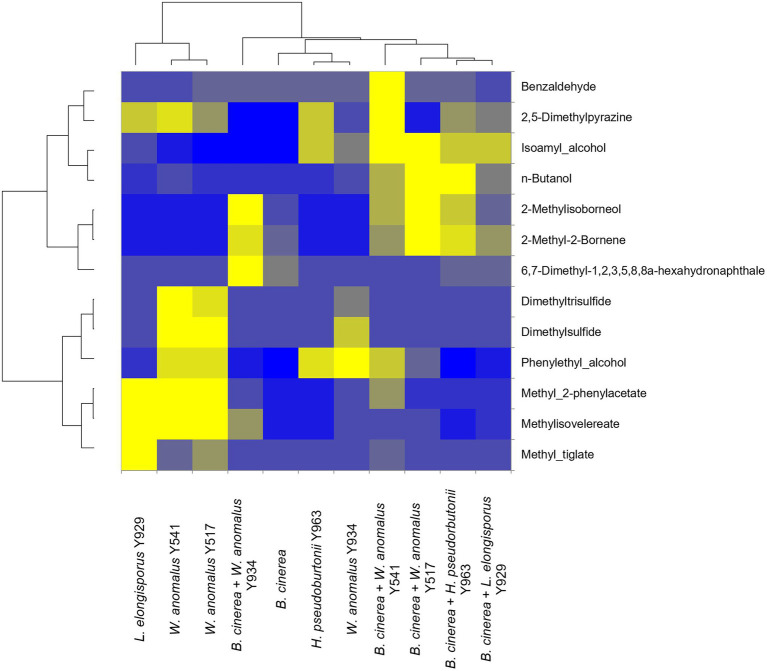
Relative fold changes coloured from blue (lowest) to yellow (highest) of volatile organic compounds produced by *Botrytis cinerea* (control) and yeast strains co-cultured with various yeasts strains. Compounds were identified using Anisole d8 (Std), comparison with mass spectra from MS NIST05 spectral library.

In order to determine whether the production of VOCs was involved in the inhibition of growth of *B. cinerea* IWBT FF1 by selected yeast strains (*W. anomalus* Y541, Y517, Y934, *L. elongisporus* Y929 and *H. pseudoburtonii* Y963), SPME-GC–MS was conducted after 10 days of incubation confrontation cultures. A total of 29 compounds were detected and identified, however, only 13 were consistent across replicates (Figure 4). These include higher alcohols, aldehydes, esters, organosulfur compounds, monoterpenes, ketones and aromatic hydrocarbons. Overall, 2-methylisoborneol, 2-methyl-2-bornene and n-butanol were enhanced when *B. cinerea* was challenged with either of the three yeasts. In addition, *W. anomalus* displayed enhanced production of dimethylpyrazine, isoamyl alcohol and benzaldehyde in the presence of *B. cinerea*. In contrast, the production of methyl-tiglate and methyl-2-phenylacetate was reduced in *W. anomalus* and *L. elongisporus* in the presence of *B. cinerea* compared to their respective monocultures (Figure 4).

### Analysis of non-volatile compounds

**Figure 5:**
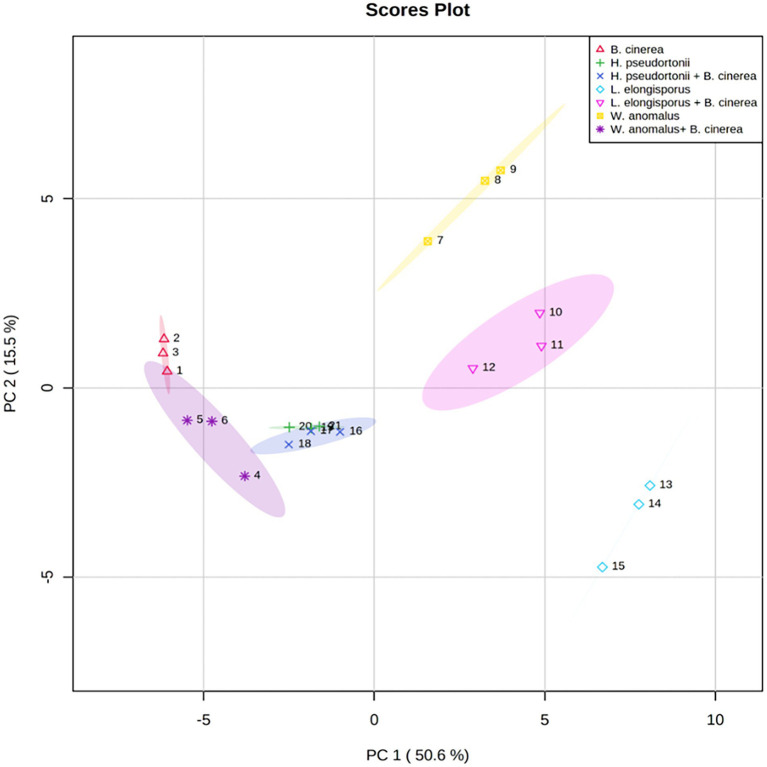
Principal component analysis score plots obtained from the non-volatile organic compounds profile of *Botrytis cinerea* and *Botrytis cinerea* co-cultured with various yeast species.

No information is available on the role of non-volatile compounds especial cyclic peptides secreted by antagonistic yeasts as mode of action, therefore only three strains (*W*. *anomalus* Y541, *H. pseudoburtonii* Y963 and *L. elongisporus* Y929) representing three species were evaluated. Non-volatile organic compounds produced by yeast in the presence of *B. cinerea* IWBT FF1 were analysed using UPLC-MS. The three selected yeasts and *B. cinerea* were co-cultured in ME broth and incubated for 5 days. Principal component analysis revealed some separation between the monocultures and mixed cultures. Although an overlap was clear between the *H. pseudoburtonii* and its mixed culture with *B. cinerea* (Figure 5). Several non-volatile compounds were identified, however, only a few cyclic peptides were differentially enhanced where the respective yeast strains were co-cultured with *B. cinerea* (Table 4). The rest of the compounds are not shown since they could not be confidently associated with fungi. The cyclic peptides were secreted by yeast strains (*W. anomalus* Y541*, H. pseudoburtonii* Y963 and *L. elongisporus* Y929). Based on the average peak intensities, l-Cyclo(leucylprolyl) was found to be the most abundant compound in *L. elongisporus* Y929 however, its intensity was decreased in the presence of *B. cinerea*. In the presence of *B. cinerea*, *W. anomalus* Y541 showed an increase in the production of Leucylproline as compared to *W. anomalus* alone. *Hyphopichia pseudoburtonii* Y963 showed an enhanced production of l,l-Cyclo(leucylprolyl), Cyclo(d-Leu-l-Trp), cyclo(l-Pro-l-Val) and Leucylproline when co-cultured when *B. cinerea*.

**Table 4:** Analysis of non-volatile organic compounds produced by *B. cinerea* (Bc), *H. pseudoburtonii* (Hp), *W. anomalus* (Wa) and *L. elongisporus* (Le).

RT (min)	[M + H]+	Compound name	Average peak height intensity						
			Bc	Hp	Le	Wa	Bc + Hp	Bc + Le	Bc + Wa
2.569	300.16891	cyclo(d-Leu-l-Trp)	17.33	63.33	476	24.33	69	99.33	20
3.335	229.15364	Leucylproline	304.66	273	287	356.33	648,33	305.66	400.33
3.965	197.12885	cyclo(l-Pro-l-Val)	298	372	633.33	366.66	467,33	326	322.33
4.624	211.14487	l,-Cyclo(leucylprolyl)	1341.33	1,224	3,388	1252.66	1,552,66	1377.33	1166.33

Compounds were analysed with UPLC-MS. Retention time (RT) and [M + H] + of tentatively identified compounds by comparison with the NIST database.

Compounds were identified and confirmed by the retention time, structures and molecular weight by comparison with NIST database.

To evaluate the effectiveness of the antagonistic yeasts on table grapes and to compare the outcomes of the *in vitro* assays, the grape bioassay in a closed airtight container was conducted. Measurements of grape deterioration are made per bunch rather than per berry. Therefore, no measures of radial inhibition were made. Efficacy of the selected yeast strains in reducing *B. cinerea* development moulds is reported in Table 5. *W. anomalus* strains *in vivo* (Table 5) showed a 90–100% reduction of *B. cinerea* growth. Soft mycelium developed in the presence of *H. pseudoburtonii* after 5 days of incubation. On grapes treated with *L. elongisporus* mycelial formation similar to that on grapes with only *B. cinerea* (control) was observed.

**Table 5:** *In vivo* test of the selected various yeast species against *Botrytis cinerea.*

Yeast strains	*B. cinerea* development
*B. cinerea*	4
*L. elongisporus* Y929	4
*L. elongisporus* Y996	4
*H. pseudoburtonii* Y963	3
*W. anomalus* Y934	2
*W. anomalus* Y541	2
*W. anomalus* Y517	2

The disease severity was evaluated by a visual score “1-to-4” (1, no visible symptoms; 2, soft rot; 3, formation of mycelium and 4, sporulation of mould).

## Discussion

Grapes and wine production worldwide suffer economic losses due to bunch rot. Mostly filamentous fungi are responsible for the decay of grapes, with *B. cinerea* (grey mould) being the most common; however, various other fungi, such as *Aspergillus* species and *Penicillium* species, can also cause the decay of grapes (Steel et al., 2013). These are widely controlled through the use of synthetic fungicides. However, growing concerns over undesirable chemical residues in the environment have spurred the exploration of non-conventional yeasts as biological alternatives (Freimoser et al., 2019; Zhang et al., 2020; Agarbati et al., 2022).

This study evaluated the antifungal activity of grape must associate yeasts against three strains of *B. cinerea*, one strain of *A. niger* and *A. alternata* and further demonstrated the possible modes of action of the selected yeast strains against *B. cinerea.* Overall, our data show wide distribution of antifungal activity across different yeast species and strains. Intraspecific variability was observed in the form of differences in the presence and absence of activity, the spectrum as well as the strength of activity. For instance, within the species *Candida oleophila*, strain Y994 could inhibit all phytopathogens tested but displayed weak glucanase and chitinase activity, while Y964 and NOVA-CH inhibited two of the pathogens and expressed strong glucanase and chitinase activity. Conversely, within the *Wickerhamomyces anomalus* and *Zygoascus meyerae* strains, similar antifungal activity spectra as well as minimum inhibitory concentrations (MIC) were observed but these differed mainly in the levels of chitinase and glucanase displayed. Furthermore, yeast strains differed in their inhibition of *B. cinerea* strains. For instance, within the *W. anomalus* species, Y517 and Y541 displayed stronger inhibition against IWBT FF1 while Y934 displayed similar inhibition levels across different *B. cinerea* strains. Intraspecific variability is a common phenomenon in yeast antifungal activity and has been reported for many species (Bleve et al., 2006; Cordero-Bueso et al., 2017; Agarbati et al., 2022). In particular, intraspecific variability antagonistic activity was observed in several strains of *W. anomalus* against two strains of *B. cinerea* and *Curvularia lunata,* a fungal plant pathogen responsible for rice dirty panicle disease (Khunnamwong et al., 2020). Yeast species such as *C. oleophila*, *W. anomalus* and *P. kluyveri* have been reported several times as potential antagonists against different moulds for various pathogens infecting different fruits such as grapes, apples, sweet cherries and strawberries (Dlamini and Dube, 2008; Parafati et al., 2015; Wang et al., 2018; Comitini et al., 2021; Agarbati et al., 2022).

Antagonistic yeasts possess several mechanisms of action including competition for nutrients and space, production of cell wall degrading enzymes, VOCs and non-volatile compounds, as well as direct mycoparasitism (Freimoser et al., 2019). In the current study, yeast strains with antifungal activity against the tested pathogens secreted chitinases and β-1,3-glucanases in the presence of *B. cinerea* with variable concomitant reduction in chitin and glucan levels demonstrated in *B. cinerea* cell walls following exposure to *H. pseudoburtonii* as well as strains of *W. anomalus*, *L. elongisporus*. The reduction in these cell wall polysaccharides could partly be due to hydrolysis by the chitinases and glucanases. Indeed, a study by Tayel et al. (2013) observed a softening of the hyphal walls and an elastic texture in *Aspergillus flavus* hyphae exposed to chitinases and glucanases from *W. anomalus* (formerly *Pichia anomala*). This hyphal softening progressed into moderate hyphal lysis and finally complete hyphal degradation. Production of β*-*1,3 glucanases and chitinases by *W. anomalus* strains as part of its arsenal against different pathogens is well known (Lutz et al., 2013; Hong et al., 2017; Chen et al., 2018; Andrea et al., 2020; Cabañas et al., 2020). However, for *L. elongisporus* and *H. pseudoburtonii* this is the first report of their antifungal activity and the potential effect of their chitinase and glucanase activity on fungal cell wall composition.

Considering the negative effects of antagonistic yeast VOCs on fungal mycelial growth, spore germination and sporulation, selected yeast strains were evaluated for the production of VOCs in the presence of *B. cinerea.* The selected yeast strains released various VOCs in the presence of *B. cinerea*. The identified volatile compounds were grouped into different chemical families, such as higher alcohols, aldehydes, esters, organosulfur compounds, monoterpenes, ketones and aromatic hydrocarbons. In particular, phenylethyl alcohol, isoamyl alcohol, n-butanol, 2,5-dimethylpyrazine, seemingly originating from yeasts, remain moderately high in the challenge experiment. Furthermore, for some yeasts such as *W. anomalus* strains, dimethylsulfide and dimethyltrisulfide could still be detected in the challenge experiment albeit at lower levels compared to the yeast monoculture. Numerous studies on antagonistic yeast such as *A. pullulans*, *P. occidentalis*, *M. guilliermondii C. tropicalis*, *S. cerevisiae* and *P. kudriavzevii* revealed similar compounds as those found in this study. These compounds have been shown to effectively inhibit spore germination and mycelial growth of *B. cinerea*, *Aspergillus* and *Penicillium* species (Arrarte et al., 2017; Meshram et al., 2017; Choińska et al., 2020; Liu et al., 2020). Production of VOCs by antagonistic yeasts has been identified as a potential mode of action against a number of pathogens including *B. cinerea*, *Colletotrichum acutatum* and various *Penicillium* species such as *P. italicum*, *P. digitatum* and *P. expansum* under both *in vitro* and *in vivo* conditions. Compounds such as phenylethyl alcohol, n-butanol, isoamyl alcohol and 2-methyl-1-propanol have been shown to supress conidia germination and mycelium growth (di Francesco et al., 2016; Yalage Don et al., 2020; Zou et al., 2022). Furthermore, a compound like dimethyltrisulfide which is not frequently reported in yeast VOCs has been shown to suppress the expression of genes involved in the biosynthesis of β-1,3-d-glucan and chitin in another fruit rotting fungus, *Colletrichum gloeosporioides* (Tang et al., 2020). These data could suggest that to suppress the growth of phytopathogens a yeast like *W. anomalus* expresses various synergistic activities including production of cell wall degrading enzymes as well as VOCs that inhibit spore germination and biosynthesis of cell wall polysaccharides. The evaluation of the efficacy of yeasts in preventing *B. cinerea* development on table grapes showed a considerable decay reduction. *W. anomalus* revealed the highest efficacy in controlling the fungal development whereby only soft rots were recorded. Since the experiment was carried out in airtight containers, the suppression of *B. cinerea* growth in the presence of *W. anomalus* could confirm the production of diverse VOCs as a mode of action among others.

The selected antagonistic yeasts were found to produce in some cases slightly higher levels of cyclic peptides such as l,l-Cyclo(leucylprolyl), cyclo-(l-Phe-l-Pro) and cyclo-(l-Phe-l-Pro). In particular, cyclo(d-Leu-l-Trp) was abundant in *L. elongisporus* monoculture and remained high in the mixed culture with *B. cinerea*. Cyclic peptides inhibit fungal development by targeting fundamental features of the fungal cell wall constituents (Hur et al., 2012). Their production by bacteria has commonly been reported and several filamentous fungi have been shown to produce these compounds. In yeast, the most commonly encountered cyclic peptide is pulcherriminic acid produced by the yeast *Metschnikowia pulcherrima* (Gore-Lloyd et al., 2019). *Glaciozyma antarctica*, a psychrophilic yeast was also shown to produce a diversity of cyclic peptides (Rosandy et al., 2017). In the current study, the majority of the cyclic peptides were detected in both monocultures and mixed cultures, suggesting that their inhibitory activity may not play a role in these interactions, but this remains to be further unravelled.

## Conclusion

Yeasts derived from grape must were found to be effective against the pathogens examined giving these yeasts potential to be developed and used as biocontrol agents. The efficacy of these yeast species can further be evaluated *in vivo* against various plant pathogens considering various environmental factors. This study largely confirmed that various mechanisms including the pathogen cell wall degradation, inhibition of cell wall polysaccharide biosynthesis as well as inhibition of spore germination through the production of VOCs may be expressed in concert yeasts interacting with filamentous fungi. Moreover, our findings open a new area for further investigation into non-VOCs of yeast origin and their contribution to antifungal activity.

## Data availability statement

The original contributions presented in the study are included in the article/Supplementary material, further inquiries can be directed to the corresponding author.

## Author contributions

EM performed the experiments, analysed the data, and wrote the manuscript. NJ, HP, and MS conceived the experiments, supervised the experimental work, assisted in the interpretation of the data, and edited the manuscript. All authors contributed to the article and approved the submitted version.

## Funding

This work is based on the research supported wholly by the National Research Foundation of South Africa (grant nos.: 118505 and 137960).

## Conflict of interest

The authors declare that the research was conducted in the absence of any commercial or financial relationships that could be construed as a potential conflict of interest.

## Publisher’s note

All claims expressed in this article are solely those of the authors and do not necessarily represent those of their affiliated organizations, or those of the publisher, the editors and the reviewers. Any product that may be evaluated in this article, or claim that may be made by its manufacturer, is not guaranteed or endorsed by the publisher.

## References

[ref1] Abbey J. A. Percival D. Abbey L. Asiedu S. K. Prithiviraj B. Schilder A. (2019). Biofungicides as alternative to synthetic fungicide control of grey mould (*Botrytis cinerea*)–prospects and challenges. Biocontrol Sci. Tech. 29, 207–228. doi: 10.1080/09583157.2018.1548574

[ref2] Agarbati A. Canonico L. Pecci T. Romanazzi G. Ciani M. Comitini F. (2022). Biocontrol of non-*saccharomyces* yeasts in vineyard against the gray mold disease agent *Botrytis cinerea*. Microorganisms 10, 200. doi: 10.3390/microorganisms10020200, PMID: 35208653 PMC8874649

[ref3] Agrawal T. Kotasthane A. S. (2012). Chitinolytic assay of indigenous *Trichoderma* isolates collected from different geographical locations of Chhattisgarh in Central India. Springerplus 1, 1–10. doi: 10.1186/2193-1801-1-73, PMID: 23526575 PMC3602610

[ref4] Andrea M. Mar M. Pic E. Meinhardt F. (2020). Killer yeasts for the biological control of postharvest fungal crop diseases. Microorganisms 8:11. doi: 10.3390/microorganisms8111680, PMID: 33138117 PMC7693540

[ref5] Arrarte E. Garmendia G. Rossini C. Wisniewski M. Vero S. (2017). Volatile organic compounds produced by Antarctic strains of *Candida sake* play a role in the control of postharvest pathogens of apples. Biol. Control 109, 14–20. doi: 10.1016/j.biocontrol.2017.03.002

[ref6] Bagheri B. Bauer F. F. Setati M. E. (2015). The diversity and dynamics of indigenous yeast communities in grape must from vineyards employing different agronomic practices and their influence on wine fermentation. South African J. Enol. Vitic. 36, 243–251. doi: 10.21548/36-2-957

[ref7] Barata A. Malfeito-Ferreira M. Loureiro V. (2012). The microbial ecology of wine grape berries. Int. J. Food Microbiol. 153, 243–259. doi: 10.1016/j.ijfoodmicro.2011.11.025, PMID: 22189021

[ref8] Bleve G. Grieco F. Cozzi G. Logrieco A. Visconti A. (2006). Isolation of epiphytic yeasts with potential for biocontrol of *Aspergillus carbonarius* and *A. niger* on grape. Int. J. Food Microbiol. 108, 204–209. doi: 10.1016/j.ijfoodmicro.2005.12.004, PMID: 16443300

[ref9] Cabañas C. M. Hernández A. Martínez A. Tejero P. Vázquez-Hernández M. Martín A.. (2020). Control of *Penicillium glabrum* by indigenous antagonistic yeast from vineyards. Foods 9, 1–22. doi: 10.3390/foods9121864, PMID: 33327475 PMC7764915

[ref10] Chen P. H. Chen R. Y. Chou J. Y. (2018). Screening and evaluation of yeast antagonists for biological control of *Botrytis cinerea* on strawberry fruits. Mycobiology 46, 33–46. doi: 10.1080/12298093.2018.1454013, PMID: 29998031 PMC6037076

[ref11] Choińska R. Piasecka-Jóźwiak K. Chabłowska B. Dumka J. Łukaszewicz A. (2020). Biocontrol ability and volatile organic compounds production as a putative mode of action of yeast strains isolated from organic grapes and rye grains. Antonie Van Leeuwenhoek 113, 1135–1146. doi: 10.1007/s10482-020-01420-7, PMID: 32372375 PMC7334268

[ref12] Comitini F. Agarbati A. Canonico L. Ciani M. (2021). Yeast interactions and molecular mechanisms in wine fermentation. Int. J. Mol. Sci. 22:7754. doi: 10.3390/ijms22147754, PMID: 34299371 PMC8307806

[ref13] Cordero-Bueso G. Mangieri N. Maghradze D. Foschino R. Valdetara F. Cantoral J. M.. (2017). Wild grape-associated yeasts as promising biocontrol agents against *Vitis vinifera* fungal pathogens. Front. Microbiol. 8:2025. doi: 10.3389/fmicb.2017.02025, PMID: 29163377 PMC5675894

[ref14] di Canito A. Mazzieri M. Foschino R. Cordero-bueso G. Vigentini I. (2021). The role of yeasts as biocontrol agents for pathogenic fungi on postharvest grapes. Foods 10:1650. doi: 10.3390/foods10071650, PMID: 34359520 PMC8306029

[ref15] di Francesco A. Martini C. Mari M. (2016). Biological control of postharvest diseases by microbial antagonists: how many mechanisms of action? Eur. J. Plant Pathol. 145, 711–717. doi: 10.1007/s10658-016-0867-0

[ref16] Dlamini N. R. Dube S. (2008). Studies on the physico-chemical, nutritional and microbiological changes during the traditional preparation of Marula wine in Gwanda, Zimbabwe. Nutr. Food Sci. 38, 61–69. doi: 10.1108/00346650810848025

[ref400] Fredlund E. Druvefors U. Ä. Olstorpe M. N. Passoth V. Schnürer J. (2004). Influence of ethyl acetate production and ploidy on the anti-mould activity of Pichia anomala. FEMS Microbiol. Lett. 238, 33–137. doi: 10.1016/j.femsle.2004.07.027, PMID: 15336413

[ref17] Freimoser F. M. Rueda-Mejia M. P. Tilocca B. Migheli Q. (2019). Biocontrol yeasts: mechanisms and applications. World J. Microbiol. Biotechnol. 35:154. doi: 10.1007/s11274-019-2728-4, PMID: 31576429 PMC6773674

[ref18] Ghosh S. Bagheri B. Morgan H. H. Divol B. Setati M. E. (2015). Assessment of wine microbial diversity using ARISA and cultivation-based methods. Ann. Microbiol. 65, 1833–1840. doi: 10.1007/s13213-014-1021-x

[ref19] Gore-Lloyd D. Sumann I. Brachmann A. O. Schneeberger K. Ortiz-Merino R. A. Moreno-Beltrán M.. (2019). Snf2 conttrol pulcherrimini acid biosynthesis and antifungal activity of the biocontrol yeast *Metschnikowia pulcherrima*. Mol. Microbiol. 112, 317–332. doi: 10.1111/mmi.14272, PMID: 31081214 PMC6851878

[ref20] Griggs R. G. Steenwerth K. L. Mills D. A. Cantu D. Bokulich N. A. (2021). Sources and assembly of microbial communities in vineyards as a functional component of winegrowing. Front. Microbiol. 12:673810. doi: 10.3389/fmicb.2021.673810, PMID: 33927711 PMC8076609

[ref21] Hong S. H. Song Y. S. Seo D. J. Kim K. Y. Jung W. J. (2017). Antifungal activity and expression patterns of extracellular chitinase and β-1,3-glucanase in *Wickerhamomyces anomalus* EG2 treated with chitin and glucan. Microb. Pathog. 110, 159–164. doi: 10.1016/j.micpath.2017.06.038, PMID: 28668604

[ref500] Hur G. H. Vickery C. R. Burkart M. D. (2012). Explorations of catalytic domains in non-ribosomal peptide synthetase enzymology. Nat. Prod. Rep. 29, 1074–1098. doi: 10.1039/c2np20025b 22802156 PMC4807874

[ref22] Jolly N. P. Varela C. Pretorius I. S. (2014). Not your ordinary yeast: non-*saccharomyces* yeasts in wine production uncovered. FEMS Yeast Res. 14, 215–237. doi: 10.1111/1567-1364.12111, PMID: 24164726

[ref23] Khunnamwong P. Lertwattanasakul N. Jindamorakot S. Suwannarach N. Matsui K. Limtong S. (2020). Evaluation of antagonistic activity and mechanisms of endophytic yeasts against pathogenic fungi causing economic crop diseases. Folia Microbiol. 65, 573–590. doi: 10.1007/s12223-019-00764-6, PMID: 31863278

[ref24] Kuchen B. Maturano Y. P. Mestre M. V. Combina M. Toro M. E. Vazquez F. (2019). Selection of native non-*saccharomyces* yeasts with biocontrol activity against spoilage yeasts in order to produce healthy regional wines. Fermentation 5:60. doi: 10.3390/fermentation5030060

[ref25] Lahlali R. Ezrari S. Radouane N. Kenfaoui J. Esmaeel Q. el Hamss H.. (2022). Biological control of plant pathogens: a global perspective. Microorganisms 10:596. doi: 10.3390/microorganisms10030596, PMID: 35336171 PMC8951280

[ref26] Liu D. Chen Q. Zhang P. Chen D. Howell K. S. (2020). The fungal microbiome is an important component of vineyard ecosystems and correlates with regional distinctiveness of wine. mSphere 5, e00534–e00520. doi: 10.1128/msphere.00534-20 32817452 PMC7426168

[ref27] Lutz M. C. Lopes C. A. Rodriguez M. E. Sosa M. C. Sangorrín M. P. (2013). Efficacy and putative mode of action of native and commercial antagonistic yeasts against postharvest pathogens of pear. Int. J. Food Microbiol. 164, 166–172. doi: 10.1016/j.ijfoodmicro.2013.04.005, PMID: 23680800

[ref28] Marsico A. D. Velenosi M. Perniola R. Bergamini C. Sinonin S. David-vaizant V.. (2021). Native vineyard non-*saccharomyces* yeasts used for biological control of botrytis cinerea in stored table grape. Microorganisms 9, 1–17. doi: 10.3390/microorganisms9020457, PMID: 33671825 PMC7926336

[ref800] Meshram V. Kapoor N. Chopra G. Saxena S. (2017). Muscodor camphora, a new endophytic species from Cinnamomum camphora. Mycosphere 8, 568–582. doi: 10.5943/mycosphere/8/4/6, PMID: 32372375

[ref29] Mewa-Ngongang M. du Plessis H. W. Hlangwani E. Ntwampe S. K. O. Chidi B. S. Hutchinson U. F.. (2019a). Activity interactions of crude biopreservatives against spoilage yeast consortia. Fermentation 5:53. doi: 10.3390/fermentation5030053

[ref30] Mewa-Ngongang M. du Plessis H. W. Ntwampe S. K. O. Chidi B. S. Hutchinson U. F. Mekuto L.. (2019b). The use of *Candida pyralidae* and *Pichia kluyveri* to control spoilage microorganisms of raw fruits used for beverage production. Foods 8:454. doi: 10.3390/foods8100454, PMID: 31590435 PMC6835701

[ref31] Nally M. C. Pesce V. M. Maturano Y. P. Rodriguez Assaf L. A. Toro M. E. Castellanos de Figueroa L. I.. (2015). Antifungal modes of action of *saccharomyces* and other biocontrol yeasts against fungi isolated from sour and grey rots. Int. J. Food Microbiol. 204, 91–100. doi: 10.1016/j.ijfoodmicro.2015.03.024, PMID: 25863340

[ref32] Ons L. Bylemans D. Thevissen K. Cammue B. P. A. (2020). Combining biocontrol agents with chemical fungicides for integrated plant fungal disease control. Microorganisms 8, 1–19. doi: 10.3390/microorganisms8121930, PMID: 33291811 PMC7762048

[ref33] Oro L. Feliziani E. Ciani M. Romanazzi G. Comitini F. (2018). Volatile organic compounds from *Wickerhamomyces anomalus*, *Metschnikowia pulcherrima* and *Saccharomyces cerevisiae* inhibit growth of decay causing fungi and control postharvest diseases of strawberries. Int. J. Food Microbiol. 265, 18–22. doi: 10.1016/j.ijfoodmicro.2017.10.027, PMID: 29107842

[ref34] Parafati L. Vitale A. Restuccia C. Cirvilleri G. (2015). Biocontrol ability and action mechanism of food-isolated yeast strains against *Botrytis cinerea* causing post-harvest bunch rot of table grape. Food Microbiol. 47, 85–92. doi: 10.1016/j.fm.2014.11.013, PMID: 25583341

[ref35] Pereyra M. M. Díaz M. A. Soliz-Santander F. F. Poehlein A. Meinhardt F. Daniel R.. (2021). Screening methods for isolation of biocontrol epiphytic yeasts against *Penicillium digitatum* in lemons. J. Fungi 7, 1–13. doi: 10.3390/jof7030166, PMID: 33669096 PMC7996618

[ref36] Piasecka-jo K. Choin R. (2020). Biocontrol ability and volatile organic compounds production as a putative mode of action of yeast strains isolated from organic grapes and rye grains. Antonie Leeuwenhoek 113, 1135–1146. doi: 10.1007/s10482-020-01420-7, PMID: 32372375 PMC7334268

[ref37] Porter T. J. Divol B. Setati M. E. (2019). Investigating the biochemical and fermentation attributes of *Lachancea* species and strains: deciphering the potential contribution to wine chemical composition. Int. J. Food Microbiol. 290, 273–287. doi: 10.1016/j.ijfoodmicro.2018.10.025, PMID: 30412799

[ref38] Pretscher J. Fischkal T. Branscheidt S. Jäger L. Kahl S. Schlander M.. (2018). Yeasts from different habitats and their potential as biocontrol agents. Fermentation 4:31. doi: 10.3390/fermentation4020031

[ref39] Rollero S. Bloem A. Ortiz-Julien A. Camarasa C. Divol B. (2018). Fermentation performances and aroma production of non-conventional wine yeasts are influenced by nitrogen preferences. FEMS Yeast Res. 18:foy055. doi: 10.1093/femsyr/foy055, PMID: 29741618

[ref40] Rosandy A. R. Abu Bakar M. Abdul Rahman N. N. Abdul Murad A. M. Muhamad A. Khalid R. M. (2017). Diketopiperazine produced by psychrophilic yeast *Glaciozyma antarctica* PI12. Malaysian J. Anal. Sci. 21, 1243–1249. doi: 10.17576/mjas-2017-2106-05

[ref41] Rossouw D. Bauer F. F. (2016). Exploring the phenotypic space of non-*saccharomyces* wine yeast biodiversity. Food Microbiol. 55, 32–46. doi: 10.1016/j.fm.2015.11.017, PMID: 26742614

[ref42] Sabaghian S. Braschi G. Vannini L. Patrignani F. Samsulrizal N. H. Lanciotti R. (2021). Isolation and identification of wild yeast from Malaysian grapevine and evaluation of their potential antimicrobial activity against grapevine fungal pathogens. Microorganisms 9:2582. doi: 10.3390/microorganisms9122582, PMID: 34946182 PMC8706701

[ref43] Sasidharan K. Soga T. Tomita M. Murray D. B. (2012). A yeast metabolite extraction protocol optimised for time-series analyses. PLoS One 7, 1–10. doi: 10.1371/journal.pone.0044283, PMID: 22952947 PMC3430680

[ref44] Schusterova D. Hajslova J. Kocourek V. Pulkrabova J. (2021). Pesticide residues and their metabolites in grapes and wines from conventional and organic farming system. Foods 10:307. doi: 10.3390/foods10020307, PMID: 33540835 PMC7913069

[ref600] Setati M. E. Jacobson D. Andongm U.-C. Bauer F. (2012). The vineyard yeast microbiome, a mixed model microbial map. PLoS One 7:e52609. doi: 10.1371/journal.pone.0052609, PMID: 23300721 PMC3530458

[ref45] Shekhawat K. Porter T. J. Bauer F. F. Setati M. E. (2018). Employing oxygen pulses to modulate *Lachancea thermotolerans*–*Saccharomyces cerevisiae* chardonnay fermentations. Ann. Microbiol. 68, 93–102. doi: 10.1007/s13213-017-1319-6

[ref46] Steel C. C. Blackman J. W. Schmidtke L. M. (2013). Grapevine bunch rots: impacts on wine composition, quality, and potential procedures for the removal of wine faults. J. Agric. Food Chem. 61, 5189–5206. doi: 10.1021/jf400641r, PMID: 23675852

[ref700] Tang L. Mo J. Guo T. Huang S. Li Q. Ning P.. (2020). In vitro antifungal activity of dimethyl trisulfide against Colletotrichum gloeosporioides from mango. World J. Microbiol. Biotechnol. 36, 1–15. doi: 10.1007/s11274-019-2781-z, PMID: 31832786

[ref47] Tayel A. A. El-Tras W. F. Moussa S. H. El-Agamy M. A. (2013). Antifungal action of *Pichia anomala* against aflatoxigenic Aspergillus flavus and its application as a feed supplement. J. Sci. Food Agr. 93, 3259–3263. doi: 10.1002/jsfa.6169, PMID: 23580136

[ref48] Varela C. Borneman A. R. (2017). Yeasts found in vineyards and wineries. Yeast 34, 111–128. doi: 10.1002/yea.3219, PMID: 27813152

[ref49] Vejarano R. Gil-Calderón A. (2021). Commercially available non-*saccharomyces* yeasts for winemaking: current market, advantages over *saccharomyces*, biocompatibility, and safety. Fermentation 7:3. doi: 10.3390/fermentation7030171

[ref50] Wang X. Glawe D. A. Kramer E. Weller D. Okubara P. A. (2018). Biological control of *Botrytis cinerea*: interactions with native vineyard yeasts from Washington state. Phytopathology 108, 691–701. doi: 10.1094/PHYTO-09-17-0306-R, PMID: 29334476

[ref51] Yalage Don S. M. Schmidtke L. M. Gambetta J. M. Steel C. C. (2020). *Aureobasidium pullulans* volatilome identified by a novel, quantitative approach employing SPME-GC-MS, suppressed *Botrytis cinerea* and *Alternaria alternata in vitro*. Sci. Rep. 10:4498. doi: 10.1038/s41598-020-61471-8, PMID: 32161291 PMC7066187

[ref52] Zhang X. Li B. Zhang Z. Chen Y. (2020). Antagonistic yeasts: A promising alternative to chemical fungicides for controlling postharvest decay of fruit. Journal of Fungi 6:158. doi: 10.3390/jof6030158, PMID: 32878102 PMC7558569

[ref53] Zou X. Wei Y. Jiang S. Cao Z. Xu F. Wang H.. (2022). Volatile organic compounds and rapid proliferation of *Candida pseudolambica* W16 are modes of action against gray mold in peach fruit. Postharvest Biol. Technol. 183:111751. doi: 10.1016/j.postharvbio.2021.111751

