# Supplementary material for: Antifungal activity of non-conventional yeasts against Botrytis cinerea and non-Botrytis grape bunch rot fungi
Source: Front Microbiol. 2022 Aug 23;13:986229. doi: 10.3389/fmicb.2022.986229 (PMC9445577; doi:10.3389/fmicb.2022.986229)
Supplement: Supplementary file 1 [file Data_Sheet_1.docx]

Supplementary Material

Supplementary material 1

Table S1. Origin and source of the yeast strains assayed in this study.

| **Species name** | **Strain** | **Source Cultivar** | **Origin (Vineyard location)** | **Year isolated** |
| --- | --- | --- | --- | --- |
| *Candida azyma* | Y979 | Sauvignon blanc | Elgin valley, SA | 2015 |
| *Candida apicola* | Y957 | Cabernet Sauvignon | Stellenbosch, SA | 2014 |
| *Candida lusitaniae* | Y833 | Cabernet Sauvignon | Stellenbosch, SA | 2011 |
| *Candida oleophila* | NOVA-CH | Chardonnay | Stellenbosch, SA | 2016 |
|  | Y964 | Cabernet Sauvignon | Stellenbosch, SA | 2014 |
|  | Y994 | Sauvignon blanc | Elgin valley, SA | 2014 |
| *Filobasidium capsuleginum* | Y938 | Sauvignon blanc | Elgin valley, SA | 2012 |
| *Hyphopichia pseudoburtonii* | Y963 | Cabernet Sauvignon | Stellenbosch, SA | 2014 |
| *Kwoniella mangrovensis* | Y535 | Cabernet Sauvignon | Stellenbocsch, SA | 2018 |
| *Lodderomyces elongisporus* | Y929 | Sauvignon blanc | Elgin valley, SA | 2012 |
|  | Y996 | Sauvignon blanc | Elgin, SA | 2014 |
| *Metschnikowia*  *chrysoperlae* | Y955 | Cabernet Sauvignon | Stellenbosch, SA | 2014 |
| *Metschnikowia bicuspidata* | Y540 | Grenache | Swartland, SA | 2018 |
| *Meira geulakonigii* | Y848 | Cabernet Sauvignon | Stellenbosch, SA | 2011 |
| *Hyphopichia burtonii* | Y951 | Cabernet Sauvignon | Stellenbosch, SA | 2014 |
| *Pichia fermentans* | Y995 | Sauvignon blanc | Elgin valley, SA | 2014 |
|  | KLBG-SB | Sauvignon blanc juice | Klosterneuburg, Austria | 2018 |
| *Pichia guilliermondii* | Y993 | Sauvignon blanc | Elgin valley, SA | 2014 |
| *Pichia kluyveri* | FRU-1 | Sauvignon blanc | Weingut Frühwirth, Austria | 2019 |
|  | NOVA-CH | Chardonnay | Stellenbosch, SA | 2016 |
|  | SIL-1 | Sauvignon blanc | Silberberg, Austria | 2019 |
| *Pichia kudriavzevii* | Y508 | Sauvignon blanc | Stellenbosch, SA | 2017 |
| *Pichia manshurica* | Y510 | Sauvignon blanc | Stellenbosch, SA | 2017 |
| *Pichia occidentalis* | BGLD-CH | Chardonnay | Burgenland, Austria | 2018 |
| *Pseudozyma fusiformata* | Y871 | Cabernet Sauvignon | Stellenbosch, SA | 2011 |
| *Wickerhamomyces anomalus* | Y517 | Sauvignon blanc | Stellenbosch, SA | 2017 |
|  | Y541 | Grenache | Swartland, SA | 2018 |
|  | Y934 | Sauvignon blanc | Elgin valley, SA | 2012 |
| *Zygoascus meyerae* | Y830 | Cabernet Sauvignon | Stellenbosch SA | 2011 |
|  | Y834 | Cabernet Sauvignon | Stellenbosch SA | 2011 |
|  | Y854 | Cabernet Sauvignon | Stellenbosch, SA | 2011 |

Liquid culture assay

Antagonistic activity of various yeasts cocultured with *B. cinerea*. The cultures were viewed under the microscope and the following images are a representative of the obtained results.

*
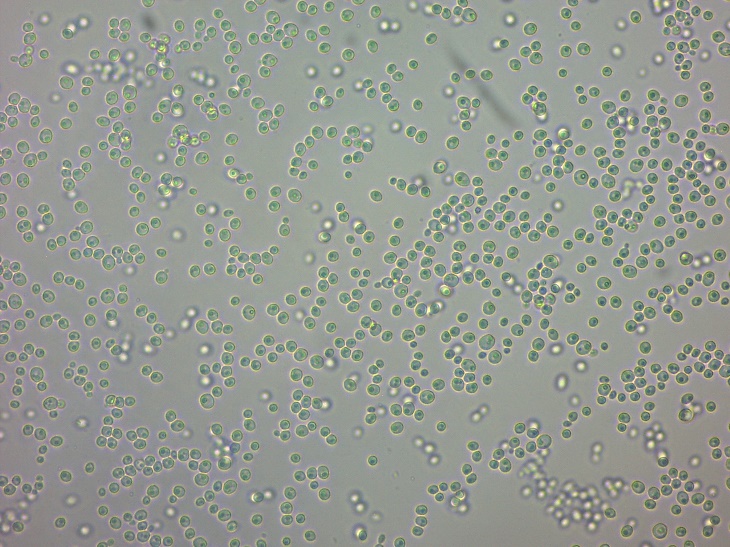

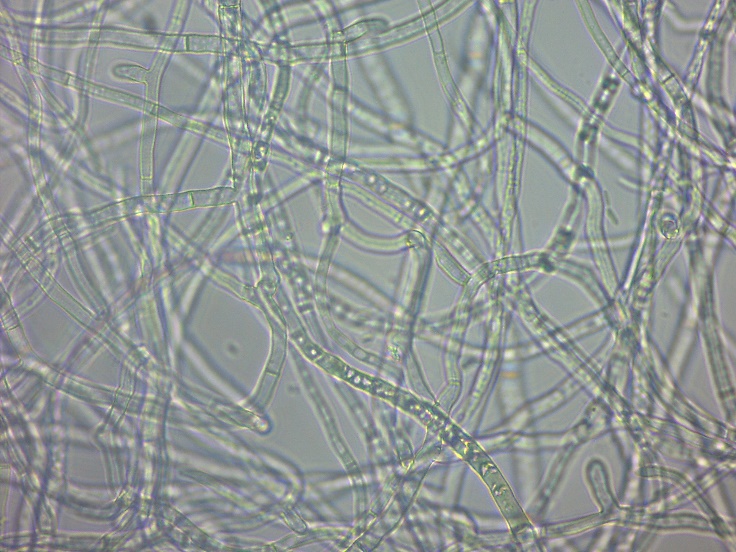
B. cinerea* ***(***formation of normal hyphae). *W. anomalus* Y541 (absence of hyphae).

*P. burtonii* Y951 ***(***limited hyphae formation***)***

*
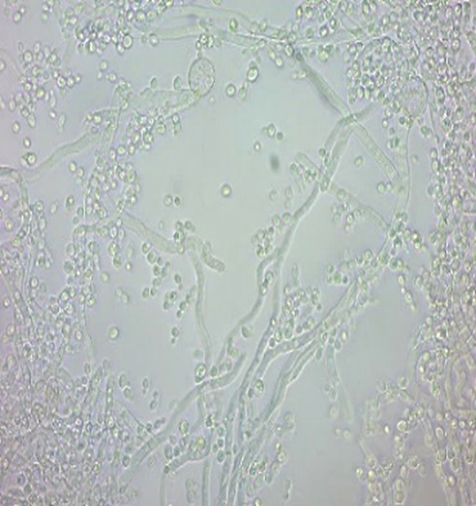
*

Figure S1. Microscopic view of *B. cinerea* (control) and *B. cinerea* treated with *W. anomalus* Y541 and *P. burtonii* Y591. Five fields on each slide were assessed and images were captured at 400x magnification.

Grape bioassay

*B. cinerea L. elongisporus H. pseudoburtonii*


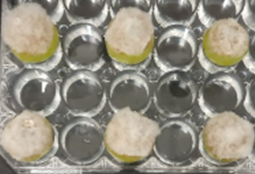

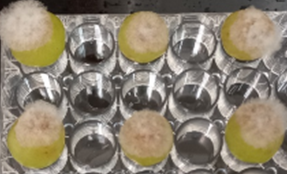

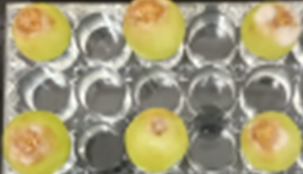


*W. anomalus*


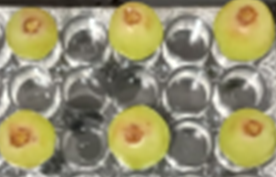


FIGURE S2. Photograph of plates showing table grapes spoilage caused by *Botrytis cinerea* and the associated antagonistic yeasts. Six grape berries per treatment were tested. Each set displayed in this figure is a representative example after 5 days incubation at 25°C.
